# Mesenchymal Stromal Cells: What Is the Mechanism in Acute Graft-Versus-Host Disease?

**DOI:** 10.3390/biomedicines5030039

**Published:** 2017-07-01

**Authors:** Neil Dunavin, Ajoy Dias, Meizhang Li, Joseph McGuirk

**Affiliations:** 1 Division of Hematologic Malignancies and Cellular Therapeutics, University of Kansas Cancer Center, 2330 Shawnee Mission Pkwy., Westwood, KS 66205, USA; adias2@kumc.edu (A.D.); jmcguirk@kumc.edu (J.M.); 2 Department of Pathology and Laboratory Medicine, University of Kansas Medical Center, 3901 Rainbow Blvd., Kansas City, KS 66160, USA; mli2@kumc.edu

**Keywords:** cell- and tissue-based therapy, exosomes, graft vs. host disease, immunotherapy, adoptive, mesenchymal stromal cells, transplantation immunology

## Abstract

After more than a decade of preclinical and clinical development, therapeutic infusion of mesenchymal stromal cells is now a leading investigational strategy for the treatment of acute graft-versus-host disease (GVHD). While their clinical use continues to expand, it is still unknown which of their immunomodulatory properties contributes most to their therapeutic activity. Herein we describe the proposed mechanisms, focusing on the inhibitory activity of mesenchymal stromal cells (MSCs) at immunologic checkpoints. A deeper understanding of the mechanism of action will allow us to design more effective treatment strategies.

## 1. Introduction

Mesenchymal stromal cells (MSCs) are multipotent progenitor cells that were first identified as a rare, non-hematopoietic cell in bone marrow that expands rapidly in vitro under standard culture conditions [1]. MSCs exist in most human tissues, with bone marrow, adipose, and perinatal tissues representing the most common sources of cells used for clinical research [2]. Regardless of the tissue of origin, all cells called “MSCs” share the following characteristics by consensus definition: they are spindle-shaped, plastic-adherent, express the markers CD73, CD90, and CD105, lack expression of hematopoietic stem cell markers, and can differentiate into chondrocytes, osteoblasts, and adipocytes under appropriate culture conditions [3]. MSCs share many phenotypic characteristics with fibroblasts and pericytes, which are closely related spindle-shaped cells in connective tissues and endothelium [2,4,5]. In general, MSCs have comparable properties regardless of the tissue source, however, there are known variations in cell surface markers, gene expression, and biological activity that depend on the source tissue [4,5,6,7].

The functions of MSCs are diverse, and several recent reviews summarize what is known. MSCs regulate immunity by interacting with innate immune cells (including macrophages, natural killer (NK) cells, and dendritic cells), and adaptive immune cells (including B and T cells), and they contribute to the restoration of immune homeostasis following infection and injury [8,9,10]. In the bone marrow, MSCs modulate the growth and differentiation of hematopoietic cells; they also contribute importantly to the three-dimensional structure and spatial orientation of cells in the stromal niche [11]. MSCs secrete an array of cytokines, chemokines, and soluble receptors that act locally and at distant sites [12,13,14]. MSCs release extracellular vesicles that contain functional proteins, lipids, and nucleic acids that are readily transferred between cells [15]. MSCs in the tumor microenvironment play a role in cancer pathogenesis [16]. Hence, MSCs are the source of intense study in human health and disease, and their biological properties suggest a role for therapeutic modulation in immunologic diseases and cancer [17].

Physicians specializing in hematopoietic cell transplantation developed an early interest in MSCs given their capacity to support the growth of hematopoietic stem cells ex vivo in co-culture [18]. With decades of experience administering cell therapies in the form of hematopoietic stem cells and lymphocytes, transplant physicians pioneered the use of MSCs as a therapeutic cell infusion. Several innate properties of MSCs led to this direction of clinical development. MSCs adhere strongly to plastic, so they are easy to separate from a mixed cell population. Once removed from their native environment, MSCs rapidly and reliably expand many-fold to clinically relevant numbers of cells. One bone marrow aspirate, which contains only 2–5 MSCs per 1 × 10^6^ mononuclear cells, can produce 1 × 10^9^ MSCs after just a few weeks of expansion and four or five subcultures. Finally, MSCs do not induce allogeneic lymphocyte responses, suggesting tolerability as a peripheral infusion.

The earliest application of MSC infusions in humans focused on hastening engraftment following stem cell transplant. A 1995 study by Lazarus and colleagues established the safety of this approach, wherein subjects with hematologic malignancies in complete remission received a one-time infusion of autologous MSCs at doses of 1 × 10^6^, 5 × 10^6^, and 50 × 10^6^ cells [19]. No subjects receiving the infusion experienced any adverse events, and MSC infusions were considered safe for further clinical development. Five years later, investigators co-infused autologous bone marrow-derived MSCs and autologous hematopoietic stem cells to hasten engraftment following high-dose chemotherapy for advanced breast cancer [20]. MSCs proved to inhibit both mitogen-stimulated lymphocytes and mixed lymphocyte reactions, which led investigators to explore their capacity to promote graft tolerance in allogeneic models of organ transplantation [18]. Bartholomew and colleagues showed that systemic infusion of both autologous and allogeneic MSCs prolonged allogeneic skin graft survival in non-human primates [21]. These early studies set the stage for subsequent safety studies using third-party human leukocyte antigen (HLA)-matched, haploidentical, and off-the-shelf unmatched MSCs for a variety of indications [22,23]. To date, dozens of studies performed over the last decade have established an excellent safety profile for both autologous and allogeneic MSC infusions over a range of cell doses [24].

Given that MSCs support stem cell engraftment, inhibit lymphocyte responses, and are safely administered as an infusion, MSCs were then considered as a potential treatment for immune complications of allogeneic stem cell transplantation [18,25]. Allogeneic stem cell transplantation (Allo-SCT) is a curative procedure for otherwise incurable hematologic malignancies. Patients receive high-dose chemotherapy and are transplanted with hematopoietic and immune cells from a human leukocyte antigen (HLA)-matched donor, thereby replacing the diseased hematopoeitic system with that of a healthy donor. It is the principal treatment for high-risk acute leukemia and the effectiveness in curing leukemias depends on the intensity of the conditioning regimen and the establishment of a graft-versus-leukemia immune response. The principal immune complication of Allo-SCT is acute graft-versus-host disease (GVHD). Acute GVHD is organ damage that arises from a failure to maintain tolerance between donor immune cells and host antigens. Acute GVHD usually presents within 100 days of transplant as an acute inflammatory process involving the skin, intestinal tract, and liver. When GVHD develops in its most severe forms (grade III or IV disease), it is associated with a nearly 40% incidence of death at one year [26]. Treatment is immunosuppression with systemic steroids, which puts patients already at risk for infection at an even higher risk. High-dose systemic steroids are the first line of immunosuppressive therapy and acute GVHD that is resistant to steroids is often fatal.

In 2004, for the first time, investigators administered a series of MSC infusions to a child with steroid-resistant acute GVHD [27]. The treatment was successful, and their report established the proof of concept that MSC infusions could be given safely and have therapeutic potential in acute GVHD. The report has since been cited in the literature over two thousand times and represents the beginning of worldwide efforts to develop MSCs as a treatment strategy for acute GVHD. To date, MSC infusions have been administered to hundreds of patients in dozens of clinical studies worldwide for the treatment of acute GVHD that is resistant to treatment with systemic steroids. These clinical experiences were recently summarized by ourselves and others [10,23,28,29]. The strongest evidence supporting efficacy is in children, and steroid-resistant acute GVHD in children is now among the leading therapeutic indications. MSC products have approvals for steroid-resistant acute GVHD in Japan, Canada, and New Zealand [30,31]. Several biotechnology companies in the United States (US) and abroad are in late stages of product development, and recently, the US Food and Drug Administration (FDA) awarded fast track designation to MSC-100-IV, an MSC formulation developed by Mesoblast, for the treatment of steroid-resistant GVHD in children. MSC-100-IV is now among a handful of MSC products that are moving closer to regulatory approval in the US.

Despite these last two decades of clinical development, the mechanisms by which MSC infusions improve GVHD outcomes remain unknown. Are their immunosuppressive and anti-inflammatory effects related to a dominant molecule, pathway, or interaction? Or are the cumulative activities of multiple factors required? Attention has recently shifted towards characterizing the immunosuppressive effects of individual soluble compounds released by MSCs for a number of reasons [13]. First, cell-to-cell contact interactions between the infused MSCs and immune cells is minimal, transient, and do not appear to be necessary to modulate immune responses. Second, MSCs are known to have a limited lifespan and essentially no long-term engraftment potential following infusion into the circulation [32]. Finally, in most clinical trials, the beneficial effects of MSCs are non-permanent and require repeat administration. Hence, the focus of this review on the immunosuppressive effects of MSCs in acute GVHD is on how specific compounds derived from MSCs modulate allogeneic immune responses. Frank and Sayegh, who wrote the accompanying commentary to the influential case report by Le Blanc and colleagues in 2004, speculated on how MSCs modulate immune responses in acute GVHD [33]. The mechanisms they proposed—co-inhibitory checkpoint activation and paracrine signaling—have continued to accumulate much support over the last decade. Indeed, several molecules that MSCs express have activity at immune checkpoints, which are a class of molecules that enhance immune signaling (co-stimulatory molecules) or inhibit immune signaling (co-inhibitory molecules). Our review will highlight these pathways and we will also consider novel concepts, such as nucleic acid interference and extracellular vesicle signaling.

## 2. Indoleamine 2,3-Dioxygenase and Tryptophan Metabolism

Indoleamine 2,3-dioxygenase (IDO) is the rate-limiting enzyme in the catalysis of tryptophan, an essential amino acid required for protein synthesis [34]. First described in 1984, IDO upregulation and the resulting catabolism of tryptophan serves as a “biostatic defense mechanism” which limits the supply of essential tryptophan to invading parasites, bacteria, and cancer cells [34,35]. In the late 1990s it was discovered that IDO-expressing cells also down-regulate immune responses and promote tolerance; hence, their biostatic properties also relate to the human immune system [36]. The mechanism by which IDO promotes tolerance is still an area of active investigation. Studies support three main pathways of IDO-mediated immunosuppression: (1) IDO activity starves T cells of tryptophan, resulting in reduced proliferation; (2) IDO produces kynurenine, O_2_ free radicals, and other derivatives that induce apoptosis in effector T cells; and (3) IDO induces tolerogenic regulatory T cells through interactions between ^−^naïve T cells and the products of tryptophan metabolism [34,37,38,39,40]. IDO is strongly upregulated in response to proinflammatory cytokines including interferon-γ (IFN-γ), interleukin-1 (IL-1), interleukin-2 (IL-2), interleukin-17 (IL-17), and tumor necrosis factor-α (TNF-α) and is an important negative feedback signal at sites of inflammation [41]. High IDO expression in fetal tissues promotes immune tolerance at the maternal–fetal interface [42]. Likewise, disorders affecting IDO signaling lead to autoimmune disease in experimental models [43]. In addition to their effects on T cells, IDO also supports the differentiation of macrophages to a more tolerogenic, interleukin-10 (IL-10)-secreting phenotype, and IDO suppresses NK-cell function [44].

Evidence suggests that IDO upregulation is an important compensatory response to acute GVHD. Jasperson and colleagues demonstrated that colon epithelial cells upregulate IDO in response to GVHD and that GVHD has increased lethality in IDO-deficient mice [45]. IDO is expressed on antigen presenting cells (APCs) in response to inflammatory signaling, leading to increased metabolism of tryptophan and greater exposure of nearby activated T cells to tryptophan degradation products. Therapeutically, a class of anticancer drugs called histone deacetylase (HDAC) inhibitors are known to induce IDO expression on APCs in a signal transducer, and activator of transcription-3 (STAT3)-dependent manner; this class of medication has been used efficaciously for the prevention of GVHD in early-phase studies [46,47,48]. In the field of cancer immunotherapy, IDO is considered one of the inhibitory immune checkpoint molecules. IDO expression by tumors is believed to be a primary mechanism of resistance to cancer immunotherapy, and pharmacologic inhibition of IDO enhances antitumor immunity in preclinical models [49]. Consequently, the use of IDO inhibitors as a single agent or in combination regimens is an area of active clinical investigation [50,51]. IDO-targeting cytotoxic T lymphocytes (CTLs) have also been used to eliminate IDO-expressing cells in the tumor microenvironment to promote antitumor immunity [52].

Human MSCs express IDO and in vitro studies first developed by Miesel and colleagues in 2004 showed that the inhibition of alloreactive T cell responses by MSCs in mixed lymphocyte reactions is dependent on IDO function [38]. In standard culture conditions, MSCs express very low levels of IDO, however, expression of IDO increases dramatically in response to inflammatory signaling from proinflammatory cytokines such as IFN-γ and TNF-α [38,41]. The upregulation of anti-inflammatory molecules following exposure to IFN-γ and TNF-α is often referred to as “priming” or “licensing” [53]. Experiments have shown that IFN-γ released by activated T cells is required for MSC-mediated immunosuppression, and IFN-γ receptor-negative MSCs lack the capacity to immunosuppress, suggesting that MSC-mediated immunosuppression by IDO signaling cannot occur without licensing [54]. In fact, most evidence suggests that MSCs fail to meet their full immunosuppressive potential unless licensed by pro-inflammatory signals, which agrees with their established role in restoring homeostasis following infection and injury. Inflammatory cytokines such as IFN-γ, TNF-α, and IL-1 are markedly increased in patients affected by acute GVHD, which potentially explains why clinical efficacy has been more conclusively demonstrated when there are mores severe manifestations of acute GVHD [55,56].

## 3. Ectonucleotidases and Adenosine Receptor Signaling

Ecto-5’-nucleotidase, known as CD73, and ectonucleoside triphosphate diphosphohydrolase-1, known as CD39, are enzymes that catalyze the conversion of nucleosides to nucleotides and phosphate. The substrate for CD73 is adenosine monophosphate (AMP), while the substrates for CD39 are adenosine triphosphate (ATP) and adenosine diphosphate (ADP) [57]. ATP is essential for a wide range of metabolic processes and serves as the principal component of energy transfer within cells. When there is massive cell damage, such as that which occurs in acute GVHD, ATP is released extracellularly where it serves as a “danger signal” or damage-associated molecular pattern (DAMP) [58]. DAMPs activate cells of the innate immune system which in turn promote T cell priming, activation, and proliferation. ATP has some additional immune-stimulatory functions: it acts as a co-stimulatory signal at the T cell receptor and drives T cell differentiation towards T helper 17 cells (T_h_17) and away from regulatory T cells [59]. By contrast, breakdown products of ATP, the adenyl nucleotides and nucleosides, play a major role in homeostatic immune suppression. Adenosine is the immunosuppressive product of AMP metabolism [60]. When adenosine interacts with the A2A receptor on T cells, effector T cells undergo anergy and regulatory T cells are produced [61]. When adenosine interacts with the A2B receptor on APCs, they take on a tolerogenic phenotype [62]. In summary, CD39 and CD73 degrade ATP to adenosine, which shifts the balance from pro-inflammatory ATP-signaling to anti-inflammatory adenosine-signaling.

Extracellular ATP concentrations increase following cell death. When ATP is recognized by the immune system, effector cells expand and produce inflammatory cytokines. Ectonucleotidases are the major regulators of extracellular ATP concentrations, and extracellular ATP is known to modulate disease activity in acute GVHD [63]. Wilhelm and colleagues showed that blocking the ATP receptor on immune cells reduces proliferation and improves survival in experimental acute GVHD [64]. Knockout of CD73 in mice leads to an exaggerated acute GVHD phenotype, and blockade of adenosine at the A2A receptor results in a similar phenotype [65,66]. Treatment of activated lymphocytes ex vivo with adenosine depletes alloreactive T cells, and adenosine reduces inflammatory cytokine production by T and NK cells [67,68]. Like IDO, ectonucleotidase activity is a well-recognized mechanism of tumor immune evasion, and CD39 and CD73 are increasingly recognized as important components of the immune checkpoint. The use of adenosine signaling inhibitors to boost tumor immune responses is an area of active clinical investigation [69]. Like IDO, CD39 is known to promote tolerance at the maternal–fetal interface [70].

MSCs express abundant CD73, and a low percentage of MSCs also express CD39. There is comparatively higher expression of CD39 on activated T and NK cells. In another example of licensing, MSCs can upregulate expression of CD39 in response to inflammatory signals [71]. MSCs also induce the expression of CD39 and CD73 on certain T cell populations, resulting in increased adenosine concentrations in T cell cocultures [72]. These observations suggest that immune cells and stromal cells cooperate to restore ATP balance following infection and injury, and further suggest that MSCs require the presence of specific immune cells to achieve maximum immunosuppressive effects [73]. CD39 is highly expressed on regulatory T cells (Tregs) and is used as a marker of this population. Like MSCs, ectonucleotidase activity and adenosine production contribute to the immunosuppressive functions of Tregs [74]. In summary, generation of adenosine from extracellular ATP by ectonucleotidases restores immune homeostasis following inflammation and injury. Massive cell death or ongoing immune attack in acute GVHD may overwhelm the capacity of this system to restore immune homeostasis. Whether or not introducing MSCs exogenously to the inflammatory environment of acute GVHD provides additional ectonucleotidase activity and shifts the ATP-adenosine balance towards anti-inflammatory signaling requires further study [75].

## 4. Programmed Cell Death Receptor and Other Inhibitory T Cell Co-Receptors

Mesenchymal cells are considered non-immunogenic because of low expression of major histocompatibility complex (MHC) class II and co-stimulatory molecules (e.g., CD80, CD86, CD40), which accounts for their safety when administered from an unmatched third party donor. In addition to IDO and adenosine, a well-recognized family of co-inhibitory ligands are also generated by MSCs: programmed cell death ligand-1 (PD-L1, also known as B7-H1) and PD-L2 (also known as B7-DC). Under normal conditions, these ligands maintain the inactivity of auto and alloreactive T cells by interactions with the PD-1 receptor (CD279) [76]. PD-1 is expressed on activated T cells, and upon engagement with PD-L1 or PD-L2, stimulatory T cell receptor signaling becomes disrupted. PD-L1 is considered IFN-γ-inducible; it is upregulated on epithelial cells, naïve T cells, dendritic cells, and tumor cells in response to inflammatory signals in the local environment [77,78]. As seen with IDO and CD39, high PD-L1 expression in fetal tissues promotes immune tolerance at the maternal–fetal interface [79]. PD-L1 expression is an important tumor escape mechanism, and therapeutic blockade of PD-1 or PD-L1 is a recent breakthrough in cancer immunotherapy. At present, monoclonal antibodies targeting PD-1/PD-L1 are a widely-used and effective treatment for a variety of cancers.

Ample evidence suggests that the PD-1/PD-L1 pathway regulates alloreactive GVHD responses following Allo-SCT. PD-1 is upregulated in alloreactive T cells, whereas blockade of PD-1 increases alloreactive T cell survival [80]. PD-1, PD-L1, and PD-L2 have increased expression in GVHD, and blocking of the signal–ligand interaction between PD-1 and PD-L1 has resulted in markedly accelerated GVHD in several studies. In work by Saha and colleagues using a mouse model of acute GVHD, rapid GVHD mortality occurred following checkpoint inhibition and was associated with increased T cell proliferation, activation, cytokine production, and reduced T cell apoptosis [81,82]. In clinical samples from human Allo-SCT recipients, high levels of PD-1 expression on allogeneic T cells measured by flow cytometry predicted poor survival following transplant [83]. In patients, blockade of PD-1 and PD-L1 has been used to effectively treat relapse after Allo-SCT; however, treatment can be complicated by severe and sometimes life-threatening acute GVHD [84,85].

MSCs express PD-L1, and expression of PD-L1 is further enhanced by IFN-γ. PD-L1 and PD-L2 have been shown to contribute to MSC-mediated T cell inhibition [86]. A contact-dependent mechanism was demonstrated by Tipnis and colleagues among others [87]. Work by Davies and colleagues showed that MSCs secrete a soluble form of PD-L1 and PD-L2 in response to IFN-γ stimulation that can inhibit T cell proliferation, establishing that MSCs do not require cell–cell contact to inhibit T cell activation by means of PD-1/PD-L1 interactions [88]. Other molecules that are necessary for co-inhibitory pathway signaling and lymphocyte activation-suppression have been implicated in GVHD, and additional co-inhibitory molecules are expressed by MSCs. One such example is the co-inhibitory molecule galectin (Gal)-3. Secretion of Gal-3 or Gal-9 into MSC culture-conditioned medium is sufficient to cause inhibition of T cell expansion [89]. Additional co-inhibitory molecules that are part of the MSC cell surface proteome include human leukocyte antigen G5 (HLA-G5) and B7-H3 [90,91].

## 5. Additional Mechanisms

While there is convincing evidence that MSCs mediate immunosuppression through inhibitory checkpoint pathways, several additional mechanisms have strong support in the literature. The following subsections highlight some of these additional pathways.

### 5.1. Prostaglandins

Prostaglandins are generated from phospholipids by cyclooxygenase-1 (COX-1) and COX-2 enzymes. Prostaglandin E2 (PGE2) has pleotropic effects on the immune system; it is generally a pro-inflammatory molecule, but its presence can result in both upregulation and downregulation of immune responses by engaging different receptors on distinct immune cell subpopulations [92]. In studies performed by Najar and colleagues, MSCs mediated lymphocyte suppression by secreting COX-1 and COX-2, resulting in increased levels of PGE2; Djouad and colleagues also showed that PGE2-mediated immunosuppression can be diminished by introducing a COX inhibitor [93,94,95]. In agreement with these findings, Chen and colleagues, in a series of co-culture experiments, showed that PGE2 blockade diminishes the immunosuppressive effects of umbilical cord-derived MSCs [96]. PGE2 secretion is induced by IFN-γ or IL-1β following mitogen stimulation or mixed lymphocyte reaction, and lack of PGE2 expression has been tied to senescence-mediated loss of immunosuppressive function [97].

### 5.2. Interleukins

Interleukins are a large family of cytokines with various activities in the immune system. MSCs both secrete interleukins and regulate the interleukin secretion of interacting immune cell populations. IL-10 secretion by MSCs results in less T_h_17 differentiation in vitro, and silencing translation of IL-10 restores differentiation [98]. Macrophages cultured in the presence of MSCs also produce IL-10, which limits antigen presentation [99]. MSCs also secrete IL-6, which can suppress dendritic cell maturation in vitro [95].

### 5.3. Growth Factors

Transforming growth factor-β (TGFβ) signaling generates Tregs and suppresses NK, B, and T cell proliferation and their cytokine production [100]. Some studies show that TGFβ may contribute to MSC-mediated immune suppression by inducing Tregs [101,102]. Vascular endothelial growth factor (VEGF) is secreted by MSC, and levels of MSC-derived VEGF can be correlated to reduced T cell proliferation [103]. TNFα-stimulated gene 6 (TSG-6) is expressed by MSCs in response to inflammation and promotes a tolerogenic macrophage phenotype; interestingly, it has been shown to predict the comparative efficacy of MSCs in vitro and in animal models [104].

### 5.4. Chemokines

Several studies have demonstrated that cooperation is needed between immune cells and MSCs to maintain immune homeostasis, and chemokines, such as chemokine ligand 9 (CXCL-9, also known as MIG) and chemokine ligand 10 (CXCL-10, also known as IP-10), may attract immune cells into the vicinity of MSCs [54]. Monocyte chemotactic protein (MCP-1) recruits T cells that undergo apoptosis after engaging the Fas ligand expressed by MSCs; apoptotic T cells then trigger macrophages to secrete TGFβ which then expands the population of Tregs [105].

### 5.5. Extracellular Vesicles

MSCs release bilayer membrane particles called extracellular vesicles (EVs) that contain functional lipids, proteins, and nucleic acids. EVs are usually sub-micron-sized, and they share elements of membrane composition with the parent cell. EVs are readily shuttled between cells. A subset of EVs commonly referred to as “exosomes” are generated through endosomal trafficking. Exosomes are 40–150 nanometers in size and are enriched for molecules found in the endosomal pathway. MSC-derived EVs are immunomodulatory, and administration of EVs recapitulates the beneficial effects of MSC treatment in murine models of acute GVHD [106]. Di Trapani and colleagues have systematically demonstrated the immunosuppressive effects of MSC-derived EVs on NK, B, and T cells [107]. Kordelas and colleagues reported a single case of successful treatment of steroid-resistant acute GVHD with MSC-derived EVs [108]. How EVs are immunomodulatory is currently being studied, but some evidence suggests that CD73-containing exosomes promote adenosine release; functional PD-L1 and galectin-1 is also enriched in the exosome fraction [109,110]. Ragni and colleagues have demonstrated that MSCs produce IL-10 mRNA that is then packaged into EVs which can be transported to cells that lack IL-10 mRNA; the receiving cell then translates the MSC-derived mRNA into functional proteins [111]. Efforts are underway to establish good manufacturing practice-compliant processes to manufacture clinical-grade MSC-derived EVs for therapeutic applications [112].

## 6. Caveats and Considerations

Given the complexity of MSC-immune cell interactions, how can we determine the principal immunomodulatory effects of MSCs? The literature describing the immunosuppressive functions of MSCs in vitro has grown rapidly, and there is now evidence and counterevidence for multiple mechanisms. For instance, several reports are in consensus regarding the role of IDO and tryptophan depletion by MSCs in the suppression of T cell proliferation, but at least one report suggests that IFN-γ licensed MSCs inhibit effector T cells independently of IDO, instead relying on upregulation of PD-L1 [86]. The complexity of interactions between immune cells, stromal cells, and the host environment in space and time may account for some of these conflicting findings. Differences in laboratory protocol and experiment design may result in study-to-study variations. Mitogen activation and mixed lymphocyte co-culture experiments are difficult to standardize owing to the number of different stimulation protocols and the need to use healthy donor immune cells. The presence of other cells (i.e., using peripheral blood mononuclear cells versus separated immune cell populations in assays) likely influences findings.

Perhaps the more important question to ask is how peripherally-administered MSCs modulate immune responses in allogeneic transplant patients with acute GVHD. Most of our knowledge regarding the immunomodulatory activity of MSCs is derived from in vitro and murine studies. There is no conclusive evidence supporting a dominant mechanism in humans with acute GVHD. However, when considering all of the established in vitro mechanisms of action, several themes emerge. First, exposure of MSCs to inflammatory signals (licensing) supports their immunosuppressive function, which aligns with their in vivo role in restoring homeostasis following infection and injury. This suggests that MSCs administered earlier in acute GVHD, during the acute phase of inflammation where they are exposed to more pro-inflammatory signals, may be more effective. Second, to some degree, immunosuppression by MSCs requires the intact function of other immune cells. In practice, MSCs are often co-administered with other immunosuppressive therapies, but it is unknown if this supports or opposes the therapeutic activity of MSCs. Third, while the principal component of acute GVHD is an allogeneic T cell response to host tissues, NK cells and macrophages also factor prominently in the pathobiology of acute GVHD, and interactions between MSCs and these other cell populations are less well characterized. Therefore, immunosuppressive mechanisms related to these additional populations in the context of acute GVHD require further study.

Some clinical studies have attempted to identify biological markers of response to MSCs in small numbers of patients. In one study, MSC-treated GVHD patients had a higher CD4^+^/CD8^+^ T cell ratio, increased frequency of Tregs, and higher levels of T cell receptor rearrangement excision circlets (TRECs), compared to pre-treatment and non-treated GVHD patients [113]. However, evidence for a causal relationship is lacking. More research on subjects who receive peripheral MSC infusions is needed, but given the regulatory considerations, high costs, complexity of Allo-SCT, and the heterogeneous manifestations of acute GVHD, it is difficult to implement a study in which biological specimens are paired with clinical outcomes. How can clinical trials be optimized to address the underlying mechanism of MSCs in acute GVHD?

## 7. Implications for Clinical Trial Design

Positive results from well-designed clinical trials are necessary for MSCs to become a widely-used treatment for acute GVHD and other disorders. Improvements in potency assays, pharmacodynamic analysis, and trial design will contribute to the robustness of future clinical studies. Each of these areas are dealt with separately in more detail below.

### 7.1. Potency Testing

MSC preparations are heterogeneous due to donor factors, source tissue, isolation method, culture conditions (i.e., seeding density, media), and number of passages. Each of these aspects are a part of the manufacturing process that can impact cell potency. Modification of cell manufacturing protocols can affect the immunosuppressive properties of MSCs. For instance, large scale culture expansion of bone marrow-derived MSCs leads to replicative senescence which reduces immunosuppressive potency, whereas IFN-γ-supplemented culture conditions lead to licensing, which increases potency [114]. Potency is commonly measured by using MSCs to suppress mitogen-activated lymphocytes or mixed lymphocyte co-cultures. These assays are difficult to standardize and have inter-assay variability. Furthermore, it is not known whether ex vivo potency correlates with therapeutic activity in acute GVHD patients. Nevertheless, lymphocyte activation–suppression is a commonly used ex vivo potency measure. Given the complexity of using cell culture-based assays, surrogate assays that correlate well with lymphocyte suppression assays have also been developed. A panel of robust and easily measured surrogate biomarkers that correspond strongly with immunosuppressive potency across several different co-culture assays may guide us towards choosing the best product. The International Society for Cellular Therapy proposes a three-pronged approach to characterization of the immunomodulatory capacity of MSCs, including: (1) quantitative RNA analysis of selected transcripts; (2) flow cytometry analysis of functionally active surface markers; and (3) protein-based assay of the secretome [115]. Array-based potency assays can take into consideration multiple factors at one time. Always choosing a product with the most immunosuppressive potency in vitro eliminates the uncertainty that variable clinical outcomes are due to product-to-product variation and potentially enhances immunosuppressive effects in vivo.

### 7.2. Biodistribution and Pharmacodynamics

Currently it is not known how to perform pharmacological monitoring after an infusion of MSCs. When a patient receives a red blood cell or platelet transfusion, the effects are easily characterized by measuring blood counts and assessing for resolving clinical symptoms. This is not so with peripherally administered MSCs, which are short-lived in the circulation and have a complex mechanism of action. Unfortunately, the principles of pharmacokinetics that are used to characterize drug therapies—absorption, distribution, metabolism, and excretion—do not apply to infusions of MSCs, which have complex fates in vivo. While MSCs are quickly cleared from the circulation, little is known about the fate of soluble factors derived from MSCs. One possible approach is to use targeted proteomics to measure important MSC-derived factors at frequent intervals following infusion. Furthermore, there are established serum biomarkers of GVHD severity (i.e., suppression of tumorigenicity 2, or ST2) that may change dynamically over the course of GVHD treatment and correlate with responses. Enzymatic activity of essential molecules can also be measured; for instance, serum kynurenine concentrations may indicate the function of IDO, and the ratio of ATP/ADP may indicate the function of ectonucleotidases. The pharmacodynamics of MSC-derived extracellular vesicles is an area for exploration but requires expertise in exosome isolation methodology. Novel attempts to explore kinetics of cell therapies such as mathematical modeling may shed light on outcomes observed in clinical studies [116]. If licensing is necessary for optimal MSC function in vivo, then the timing of MSC administration should coincide with the timing of highest concentrated inflammatory cytokines; thus, host factors may contribute importantly to therapeutic efficacy and can be measured at intervals. If the presence of other immune cells is required, then immune cell populations can be measured as well.

### 7.3. Power and Endpoint Selection

To establish efficacy, in general, clinical trials must enroll adequate patient numbers, randomize between an intervention and standard therapy, and demonstrate substantial improvement in a clinically-meaningful primary endpoint. Translational research that is performed in the context of such studies is powerful because research hypotheses can be measured against important clinical outcomes, such as “GVHD-free, relapse-free survival” in the case of Allo-SCT [117]. Serum biomarkers, such as ST2, have been shown to strongly predict poor GVHD outcomes, and biomarker-guided treatment strategies may lead to a larger effect size by excluding less severe forms of acute GVHD. Optimizing cell potency and dosing schedules may heighten treatment effects. Smaller studies can show conclusive benefit when the magnitude of the therapeutic effect is large, which is recognized by regulatory agencies in the US.

In conclusion, future studies of MSCs in acute GVHD will lead to stepwise improvements in product selection, timing, dose, frequency, and method of administration, which hopefully will result in more effective treatment strategies for patients suffering from acute GVHD. The optimization of MSC infusion therapy in acute GVHD may generate data supporting their best use in other diseases of immunity and inflammation. Despite substantial progress, how MSCs module immune responses when administered peripherally during an acute GVHD episode remains to be elucidated. Clinical trials should adhere to good design principals and include correlative studies that thoughtfully consider this important biological question.
